# The genetics of human obesity

**DOI:** 10.1111/nyas.12020

**Published:** 2013-01-29

**Authors:** Qianghua Xia, Struan FA Grant

**Affiliations:** 1 Division of Human GeneticsPhiladelphia, Pennsylvania; 2 Center for Applied Genomics, The Children's Hospital of Philadelphia Research InstitutePhiladelphia, Pennsylvania; 3 Department of Pediatrics, Perelman School of Medicine, University of PennsylvaniaPhiladelphia, Pennsylvania

**Keywords:** obesity, genetics, monogenic, GWAS, human

## Abstract

It has long been known that there is a genetic component to obesity, and that characterizing this underlying factor would likely offer the possibility of better intervention in the future. Monogenic obesity has proved to be relatively straightforward, with a combination of linkage analysis and mouse models facilitating the identification of multiple genes. In contrast, genome-wide association studies have successfully revealed a variety of genetic loci associated with the more common form of obesity, allowing for very strong consensus on the underlying genetic architecture of the phenotype for the first time. Although a number of significant findings have been made, it appears that very little of the apparent heritability of body mass index has actually been explained to date. New approaches for data analyses and advances in technology will be required to uncover the elusive missing heritability, and to aid in the identification of the key causative genetic underpinnings of obesity.

## Introduction

Obesity has long been recognized as a condition that affects one's well-being. Indeed, the ancient Greek physician Hippocrates was first to realize the association of obesity with disease, noting that “sudden death is more common in those who are naturally fat than in the lean.”^1^ However, others reject the term *disease* because such a label “medicalizes” a huge population of people. The phenotype of obesity has become an increasingly serious health issue worldwide, particularly in the more affluent industrialized countries, where the United States currently tops the rankings for most obese nation. This a major cause for concern, as obesity, plus the development of insulin resistance,^2^,^3^ has been associated with many common medical conditions, such as type 2 diabetes, hypertension, cardiovascular disease, stroke, and physical disabilities.^4^

According to the Centers for Disease Control and Prevention (CDC), there has been a steady increase in the rates of obesity in the United States over the past two decades, rising from 19.4% in 1997, to 24.5% in 2004, 26.6% in 2007, 33.8% in 2008, and 35.7% of adults in 2010. Obesity in children is also in excess of 17% in the United States; indeed, approximately 70% of obese adolescents grow up to become obese adults,^5^–^7^ which in turn increases their overall mortality later in life.^8^ In 2010, the medical costs of treating people for overweight and obesity were estimated to be $72 billion and $198 billion, respectively, thus posing a huge financial burden on the healthcare system and therefore on the U.S. economy.^9^

The World Health Organization (WHO) defines overweight and obesity as abnormal or excessive fat accumulation that may impair health.^10^ The most commonly used measure of obesity, due to its ease of ascertainment, is body mass index (BMI), which is defined as a person's weight in kilograms divided by the square of the person's height in meters (kg/m^2^). Although BMI is a comparative index and does not provide a direct measure of body fat content, many studies have shown that this metric correlates well with fat content in the vast majority of people. Adults with a BMI between 25 and 30 are considered overweight, and those with a BMI greater than or equal to 30 are considered obese. However, in the context of childhood, BMI is both age and sex dependent; thus a BMI-for-age percentile ranking is generally used to ascertain this metric in the pediatric setting; a BMI from the 85th to 95th percentile is considered overweight, while BMI equal to or greater than the 95th percentile is considered obese.^11^,^12^ By late adolescence, these percentiles begin to mesh with the adult definitions, where the 95th percentile represents a BMI of approximately 30 kg/m^2^.^13^

Despite the current obesity epidemic, data supporting the use of existing pharmacological agents are far from convincing. For instance, sibutramine^14^ exhibits side effects, including increases in heart rate and blood pressure,^15^ while orlistat is associated with gastrointestinal side effects that require fat-soluble vitamin supplementation and monitoring.^16^,^17^ Thus, there is great opportunity to identify targets for more efficacious and more specific therapeutic intervention. Understanding the genetics of BMI determination and obesity holds great promise for delivering many more such targets in the relatively near future.

## Evidence for a genetic component to obesity

The pathogenesis of obesity is clearly complex, involving multiple interactions among behavioral, environmental, and genetic factors. The rising prevalence of obesity can be partially attributed to highly calorific food intake and the relatively sedentary lifestyle of modern times;^18^,^19^ indeed, what is now considered a disease could well have been an advantage in more primitive times when food was less available, and when high energy expenditure through physical activity was a way of life. Thus those with a so-called thrifty phenotype had a survival advantage due to a more efficient use of calories.

Accumulating evidence has strongly implicated a genetic component playing an important role in the risk of becoming obese.^18^,^19^ Twin studies have been a much used model to allow the assessment of the genetic component to a given trait due to the fact that monozygotic (MZ) twins are genetically identical, while nonidentical dizygotic (DZ) twins share only 50% of their genetic material; the concordance for fat mass among MZ twins has been reported to range from 70–90%, while in DZ twins it is 35–45%.^21^–^23^ Although such data provide strong evidence for a genetic component to obesity, the explanation for the wide range in estimates of heritability derived from twin studies is largely dependent on how the studies are performed; for example, in one of Stunkard's seminal studies, heritability of 77% for BMI was found to increase to 84% at a 25-year follow-up in a sample of 1,974 MZ and 2,097 DZ male twin pairs.^24^

Further evidence comes from adoption and family studies, which have shown a strong correlation between the BMI of the adoptees and biological parents but not with adoptive parents.^25^ In addition, it has been shown that while there is no association between BMI of nonidentical twins separated at birth, there is a significant relationship for identical twins raised apart.^26^ Differences in prevalence of obesity among racial/ethnic groups also provides genetic insight; for example, a prevalence of 35% or less in Caucasian and Asian populations to 50% or more in Pima Indians and South Sea Island populations.^27^

Such findings strongly support the concept that genes play a central role in the determination of BMI and, consequently, in the pathogenesis of obesity. However, it is has generally proved challenging to identify the specific underlying genetic cause of obesity, and this is due to the complex interactions involved in the regulation of adiposity.

## Monogenic obesity

Studies of single gene disorders that exhibit the features of obesity have been a useful model to get the first insights into the genetics of the trait, with initial studies being carried out in rodent models being somewhat fruitful.

The *ob/ob* mutant mouse strain was described as early as the 1950s,^28^,^29^ exhibiting excess adipose tissue along with impaired reproductive behavior^30^ and weighing approximately three times more than normal mice by maturity; however, the *ob* mutation remained elusive for many years; but in 1994 positional cloning was used to identify and characterize the gene.^31^ A 16-kDa secreted protein product is encoded by the leptin gene, derived from the Greek root *leptos*, meaning thin.^32^ Interestingly, in another strain of severely obese mice, *db/db*, a mutation in the leptin receptor gene was identified.^33^

Leptin is primarily expressed and secreted by adipocytes, while its receptor is primarily expressed in the hypothalamus^33^ and plays a major role in the regulation of food intake, energy balance, and body weight in both rodents and humans. In addition, it has been shown in humans that serum leptin concentrations are positively correlated with obesity-related traits.^34^ After the identification of the leptin gene (*lep*) in mice, leptin mutations were also found in human patients, including a homozygous reading frame shift, a series of missense mutations and a number of polymorphisms that were associated with obesity.^35^–^37^

In addition, homozygotes for a mutation in the human leptin receptor gene resulting in the skipping exon16, which produces a truncated protein lacking both the transmembrane and the intracellular domains, show impaired growth hormone secretion, early-onset morbid obesity, and failure of pubertal development.^38^ Furthermore, common leptin receptor polymorphisms have been shown to be associated with obesity in Caucasians.^39^

Proopiomelanocortin (POMC) is produced by the hypothalamus and plays a role in feeding behavior, with *POMC* expression being positively regulated by leptin.^40^ A frame shift mutation in the *POMC* gene has been reported to cause loss of function, resulting in early-onset obesity, adrenal insufficiency, and red hair pigmentation,^41^ while other mutations in *POMC* associated with severe and early onset obesity have been described in two unrelated German individuals.^42^ In addition, a R236G mutation in *POMC* disrupts proteolytic cleavage sites between the POMC-encoded β-MSH and β-endorphin peptides, and produces an aberrant fusion protein with lower binding affinity to the melanocortin-4 receptor(MC4R),^43^ a seven-transmembrane G-protein-linked receptor expressed in the hypothalamus.

Furthermore, a single patient with severe early onset obesity was reported to have compound heterozygote mutations in the proprotein convertase subtilisin/kexin type 1 (*PCSK1;* also known as *PC1*) gene,^44^ a key component in the proteolytic processing of POMC;^45^ more recently, common nonsynonymous variants in *PCSK1* have also been shown to confer risk of obesity.^46^ Indeed, biochemical experiments with N221D demonstrate that its enzyme activity is reduced by 10%,^47^ making it an attractive drug target going forward.

MC4R plays an important role in controlling leptin's effects on food intake and body weight,^48^ and mice with disruption of the *MC4R* gene are severely obese.^49^ Multiple nonsense and missense mutations have now been identified in the human form of this gene, and these mutations are strongly associated with many obesity related traits.^50^,^51^ Indeed, *MC4R* is considered the first locus at which mutations are associated with a rare form of dominantly inherited morbid human obesity, and was the best known cause of human obesity before the era of genome wide association studies (GWAS).

It has been shown that brain-derived neurotrophic factor (BDNF) and its receptor, tyrosine receptor kinase B (encoded by *NTRK2*) regulate eating behavior and energy balance. Mice with the conditional knockout of *BDNF* develop obesity and hyperactivity,^52^–^54^ with subsequent human studies showing a chromosomal inversion leading to the loss of one functional copy of *BDNF* in an 8-year-old girl; this resulted in increased food intake, severe early-onset obesity, hyperactivity, and cognitive impairment.^55^ In addition, a Y722C missense variant in *NTRK2* has been reported in an 8-year-old boy with severe obesity and impaired memory.^56^

*SIM1* is the homologue of *Drosophila melanogaster* single-minded (sim), a helix-loop-helix-PAS domain transcription factor. Sim plays an important role in the differentiation of central nervous system (CNS) midline cells within the ventral neurogenic region in *Drosophila*.^57^ In mice, *Sim1* heterozygousity leads to early-onset obesity, with increased linear growth, hyperinsulinemia, and hyperleptinemia,^58^ while haploinsufficiency of *SIM1* has been reported to cause severe early-onset human obesity due to a balanced translocation between chromosomes 1p22.1 and 6q16.2.^59^

The above data indicate that recent studies of genetic syndromes of obesity in rodents have provided substantial insights into the underlying mechanisms involved in energy homoeostasis and have revealed key loci that operate in the human equivalent of the disease. In recent years, however, research has begun to directly identify human disorders of energy balance that arise from defects in other loci,^60^ including Prader–Willi,^61^ Alström's,^62^ and Bardet–Biedl syndromes.^63^–^65^

## Family studies for common obesity

Genome-wide linkage scans in families with the more common forms of obesity than the monogenic forms described above have yielded several loci, but the genes within these loci have generally remained elusive.

In order to identify as yet unknown genes involved in obesity, investigators have attempted to scan the entire genome to search for linkage between polymorphic markers and disease-related phenotypes. Using genetic makers evenly located across the whole genome, researchers can determine where specific loci are shared more often among affected individuals within and between families than would be expected by chance. This method has been successful in mapping the monogenic versions of the disease while some quantitative trait loci, such as for height or body weight, have been mapped, including the genes encoding the glutamic acid decarboxylase enzyme (*GAD2*), ectonucleotide pyrophosphatase/phosphodiesterase1 (*ENPP1*) and solute carrier family 6 (amino acid transporter), member 14 (*SLC6A14*);^66^–^70^ however, some of these loci still require further validation by independent groups.

## Candidate gene studies for common obesity

Before GWAS, most candidate gene studies of common obesity looked at variants in genes already implicated in the rarer, monogenic forms of the disease. Overall, such studies to date have achieved only limited success in identifying genetic determinants of obesity, primarily due to their general reliance on a suspected disease-causing gene whose identity derives from a particular biological hypothesis on the pathogenesis of obesity. Thus, since the pathophysiological mechanisms underlying obesity are generally still unknown, continued use of the hypothesis-driven candidate gene association approach is likely to identify only a relatively small fraction of the genetic risk factors for the disease.

Common variants in the genes encoding leptin gene and the leptin receptor have been shown to be associated with BMI and obesity in several populations.^71^–^74^ For example, adiponectin, a hormone that plays a key role in regulation of glucose and fatty acids^75^ and has reduced levels in obesity and type 2 diabetes,^76^ has been found to be genetically associated with these diseases around the world.^77^–^81^ In addition, association studies of various nominated candidate genes have also implicated the genes encoding such factors as the cannabinoid receptor 1 (*CNR1*), dopamine receptor 2 (*DRD2*), serotonin receptor 2C (*HTR2C*), and *SLC6A4,*^82^–^86^ but the most replicated of them is the Pro12Ala substitution in the peroxisome proliferator-activated receptor-gamma(*PPARγ*) gene, which has been extensively associated with both obesity^87^ and type 2 diabetes.^88^ These studies support the conclusion that obesity is a complex disease influenced by many genes with small effect size; thus there is a great need for nonhypothesis genome wide approaches.

## Initial GWAS approaches: uncovering the fat mass and obesity-associated gene

To date, family-based linkage analyses have had limited success in identifying genes contributing to obesity due to a number of reasons, including the issue of bandwidth, where this approach is generally poor at detecting common genetic variants that confer only modest risk.^89^,^90^ The candidate gene association study is limited to known biological mechanisms (as described above) so in order to uncover novel loci contributing to the pathogenesis of the trait, one requires a more global and nonhypothesis approach.

The GWAS approach is a high-throughput methodology that allows geneticists to scan a dense set of single nucleotide polymorphism (SNP) makers (∼0.1–5 million SNPs) spanning across the entire human genome in an unbiased manner, using powerful statistical methods to study associations between a given disease phenotype and a representation of all common variation in the genome. These SNPs are by no means considered causal; rather they operate as tag-SNPs that capture the common haplotypic variation in a given region of the human genome. If a given tag-SNP is associated with a particular trait, it signifies a region of typically a few hundred kilobases where a causative variant should reside. The success of the GWAS approach was made possible by the HapMap project, a large-scale effort that has extensively characterized human sequence variation,^89^–^91^ in addition to the development of high-density genotyping arrays,^92^,^93^ which allow one to score the alleles for a large number of SNPs in parallel across the genome.^94^,^95^

Owing to the non-hypothesis-driven nature of GWAS, this technique can be used to uncover new insights into the biology of a given phenotype without any prior knowledge of function; that is, one simply starts with a blank canvas and the genetics leads to the most strongly associated regions of the genome. GWAS have turned out to be a very successful approach, with many new loci implicated in complex traits; most crucially, once robust associations are detected, there is general consensus among fellow researchers—a situation that sharply contrasts with that in the candidate and linkage analysis eras (see the continually updated NIH Catalog of Published Genome-Wide Association Studies at http://www.genome.gov/gwastudies).

Like most diseases, BMI and obesity GWAS have implicated multiple loci in the past six years. Insulin-induced gene 2 (*INSIG2*) was the first locus to be reported in a GWAS of obesity;^96^ however, replication efforts yielded very inconsistent results.^97^–^101^ On the other hand, the fat mass and obesity-associated (*FTO*) gene is widely considered the first unequivocal and most robust common obesity–susceptibility locus found to date.^102^ The Wellcome Trust Case Control Consortium, in fact, uncovered *FTO* in 2007 when they carried out a GWAS for type 2 diabetes,^103^,^104^ identifying a cluster of SNPs in the first intron of the gene; however, they soon realized that the primary phenotype was actually obesity, due to the fact that the association between *FTO* variants and type 2 diabetes was ablated when there was correction for BMI. Many follow-up studies in children and adults from different populations, including European, African, and Asian ancestries, have confirmed the initial findings on FTO.^105^–^109^ Most studies are focused on the rs9939609 *FTO* single nucleotide polymorphism (SNP) that shows the strongest association with BMI in Caucasians; but more recently the variant SNP rs3751812 has been proposed to capture the *FTO* association with obesity in both European and African ancestry,^109^,^110^ even though it may not be the optimal tag-SNP for populations of European ancestry.

Although the effect size of variation at the *FTO* locus is not comparable to the more impactful rare variants in the monogenic forms of the disease, it remains the most established association with common obesity to date, primarily due to its high frequency in the population, where the minor allele frequency of the rs9939609 allele ranges from 38% to 44% in Caucasians. More specifically, Caucasian heterozygotes for the rs9939609 risk A allele have a higher BMI than the homozygotes for the protective T allele, with the difference representing approximately 0.36 kg/m^2^. Its role in other ethnicities have still to be fully elucidated.

The functional mechanism of FTO in obesity remains far from clear. The *FTO* gene in humans harbors nine predicted exons and is located in a 410,507 bp genomic region on chromosome 16. Comparative genome studies indicate that the *FTO* homologue can be found in vertebrates and marine algae, but not in plants, fungi, and invertebrate animals.^111^ Murine studies indicate that *FTO* gene expression is most abundant in the brain—particularly in hypothalamic nuclei where energy balance is influenced—and that *FTO* mRNA levels in the arcuate nucleus are regulated by feeding patterns.^112^ Following the report of the GWAS association, the *FTO* protein product was soon characterized to be a 2-oxoglutarate-dependent nucleic acid demethylase, belonging to the (2OG) oxygenases AlkB family of proteins.^112^ In both bacteria and mammals, AlkB is a DNA repair enzyme that catalyzes Fe(II)- and 2OG-dependent demethylation of damaged DNA substrates.^113^ Furthermore, a recent study indicated that FTO also demethylates *N*6-methyladenosine (m6A) residues in nuclear RNA.^114^ These results demonstrate a key role for FTO in nucleic acid repair or modification; however, it is still far from clear what the connection between this mechanism and obesity actually is.

*FTO* was originally identified through a deletion in the murine form of the gene related to fused toes (*Ft*); indeed, homozygosity of *Ft/Ft* leads to embryonic death. Following the GWAS obesity identification of *FTO*, two mouse models were used to investigate its biological function *in vivo*. Homozygous *Fto^−/−^* mice show postnatal growth retardation, significant reduction in adipose tissue, and addition of lean body mass,^115^,^116^ and mice with an I367F substitution share a similar phenotype with the *Fto^−/−^* mice.^117^ These results indicate that FTO plays a role in controlling food intake, energy expenditure, and energy homeostasis.

Follow-up human studies have been less clear when they sought rare variants in the *FTO* gene that influence obesity risk. For example, a homozygous R316Q point mutation, identified in a large Palestinian Arab consanguineous family, abolishes FTO function (because the highly conserved arginine 316 is required for 2-oxoglutarate binding, a prerequisite for enzyme activity). Patients with this mutation exhibit severe growth retardation and multiple malformations.^118^ In a second group, a French sequencing effort in Caucasians reported a set of different exonic mutations in *FTO*, also resulting in loss of function. However, neither of the *FTO* variants were conclusively associated with obesity.^119^ A similar observation was subsequently reported in African Americans.^120^

## Meta-analyses of GWAS datasets

Once investigators have fully analyzed their GWAS datasets collaborative efforts, meta-analyses can be performed to achieve statistical power to uncover additional loci; indeed, such analyses aide in overcoming multiple testing of hundreds of thousands of markers for which the threshold of significance is in the range of 5 × 10^−8^, in order to rise above statistical noise. In addition, the advent of imputation,^121^ where one computationally increases the number of SNPs considered by inferring frequency based on neighboring variant frequencies, has allowed for more extensive appraisal of association with the trait across the genome.

However, it has turned out that newer BMI/obesity-associated loci have substantially smaller effects than does *FTO,* even though they provide further insight into the biology of the BMI/obesity phenotype. In the case of BMI and obesity, the first large-scale multicenter meta-analysis of genome-wide association studies from four European populations, consisting of 16,876 individuals, observed the *FTO* association and discovered a common variant near the already established *MC4R* gene.^122^ It subsequently became clear that the *FTO* locus harbors both common and rare variants that contribute to various forms of obesity.

A subsequent larger meta-analysis carried out by the Genetic Investigation of ANthropometric Traits (GIANT) consortium^123^] in Caucasians (*n* > 32,000) uncovered six more genes: transmembrane protein 18 (*TMEM18*), potassium channel tetramerization domain containing 15 (*KCTD15*), glucosamine-6-phosphate deaminase 2 (*GNPDA2*), SH2B adaptor protein 1 (*SH2B1)*, mitochondrial carrier 2 (*MTCH2*), and neuronal growth regulator 1 (*NEGR1*). Five of these genes were also observed in a large GWAS reported from Iceland that also reported novel loci on 1q25, 3q27, and 12q13,^124^ in addition to showing association with a common variant in the already established *BDNF* locus.^125^

The most recent, expanded GIANT meta-analysis, bringing in further genome wide genotyped cohorts totaling 249,796 individuals, uncovered 32 BMI-associated loci associated with BMI.^126^ Ten loci were already known to be associated with BMI, 4 were known in the context of weight and/or waist–hip ratio (*SEC16B*, *TFAP2B*, *FAIM2*, *NRXN3*) and 18 were novel (*RBJ-ADCY3-POMC*, *GPRC5B-IQCK*, *MAP2K5-LBXCOR1*, *QPCTL-GIPR*, *TNNI3K*, *SLC39A8*, *FLJ35779-HMGCR*, *LRRN6C*, *TMEM160-ZC3H4*, *FANCL*, *CADM2*, *PRKD1*, *LRP1B*, *PTBP2*, *MTIF3-GTF3A*, *ZNF608*, *RPL27A-TUB,* and *NUDT3-HMGA1*). In addition, the array revealed a common copy number variation (CNV), in the form of a 21kb deletion, 50kb upstream of *GPRC5B*.

## GWAS-related investigations in other ethnicities

The vast majority of BMI and obesity GWAS investigations published to date have been executed in of populations of European origin. This is primarily due to the relatively low haplotypic complexity of Caucasian genomes as a consequence of the relative newness of this ethnicity, but also to get around admixture concerns. Large-scale GWAS studies of BMI and obesity in populations of African ancestry are still to be reported, as there are many challenges related to much higher haplotypic complexity and particular issues regarding admixture specific to African Americans.

On the other hand, studies in East Asians have started to appear, with the most prominent being a recently published meta-analysis of multiple cohorts.^127^ Using 27,715 individuals in the discovery stage, followed by *in silico* and *de novo* replication studies in a further 37,691 and 17,642 individuals, respectively, revealed a number of loci. Seven previously identified loci were detected (*FTO*, *SEC16B*, *MC4R*, *GIPR*-*QPCTL*, *ADCY3*-*DNAJC27*, *BDNF,* and *MAP2K5*) and three novel signals were uncovered, *CDKAL1*, *PCSK1,* and *GP2*. Additional evidence, albeit not significant, was also seen for the loci *GNPDA2*, *TFAP2B,* and *PAX6*. An additional East Asian study also implicated *CDKAL1* and *KLF9* as BMI loci;^128^ thus, studying another ethnicity revealed additional loci that were not readily detectable in Caucasians, primarily due to haplotypic structure differences. Another example of this is with the refinement of the *TCF7L2* association with type 2 diabetes^129^ down to a single, presumably causative, SNP working with a West African patient cohort.^130^ With respect to BMI and obesity, the role of the *FTO* locus in populations of African ancestry was initially less obvious;^108^,^131^ however, from large cohort studies^109^,^110^ it is becoming increasingly clear that rs3751812 allele is a good proxy for the underlying association in Africans and Caucasians.

## Testing adult-discovered loci in children

Childhood obesity is considered to have reached epidemic levels in developed countries, which raises the obvious question, How do the loci described above in adult studies function in childhood?

A recent query of all 32 loci reported in the recent GIANT meta-analysis of adult BMI in a pediatric European American dataset, consisting of 1,097 childhood obesity cases (defined as BMI ≥ the 95th percentile), together with 2,760 lean controls (defined as BMI < the 50th percentile), aged between 2 and 18 years old^132^ found evidence for association with nine of these loci, namely at *FTO*, *TMEM18*, *NRXN3*, *MC4R*, *SEC16B*, *GNPDA2*, *TNNI3K*, *QPCTL,* and *BDNF*. Overall, it was clear that 28 of the 32 loci showed directionally consistent effects to that of the adult BMI meta-analysis. It is therefore clear that the bulk of these obesity-conferring variants are operating early on in life, suggesting that intervention in childhood is warranted.

A similar number of loci have reported from GWAS investigations of type 2 diabetes; however, the mechanism through which they exert their effect is far from clear. The investigators checked if any of these loci could be influencing risk of obesity in childhood and therefore setting those same individuals up for a higher risk of type 2 diabetes later in life. A single locus did show up, where the same variant in *IDE-HHEX* that increases the risk of developing type 2 diabetes is also associated with increased pediatric BMI,^133^ thus shedding some light on the mechanism of action by this particular genomic region.

## Loci specifically identified in childhood obesity GWAS analyses

It is widely considered that distillation of the genetic component for some complex traits is easier in children, where the relative environmental exposure time is far less. So if a child is expressing a specific trait, it is more likely due to genetic factors, as the environmental component is substantially lower.

Although multiple studies have mapped genes involved in monogenic forms of obesity, which generally express themselves early on in life (see above), less has been done in the context of more common forms of obesity.

The first such study to fully address a GWAS of childhood obesity was published in 2010. The study involved the joint analysis of GWAS data from French and German study groups^134^ for early-onset extreme obesity, defined as BMI ≥ the 99th percentile. They identified two loci, at *SDCCAG8* and *TNKS/MSRA*, respectively. However, their effect was at most marginally associated in the most recent adult GIANT meta-analysis,^126^ suggesting that their effect is limited to an extreme pediatric setting.

More recently, a large-scale meta-analysis was reported of 14 existing GWAS datasets, where the definition of the disease was less extreme, that is, BMI ≥95th percentile, in order to address the more accepted definition of common childhood obesity^135^ (5,530 cases and 8,318 controls defined as <50th percentile of BMI). In addition to detecting seven known loci (*FTO*, *TMEM18*, *POMC*, *MC4R*, *FAIM2*, *TNNI3K,* and *SEC16B*), this study also revealed two novel loci, namely rs9568856 near *OLFM4* and rs9299 within *HOXB5*. When investigated within the latest GIANT adult meta-analysis of BMI, the two loci were both significantly associated with the trait and in the same direction, but were below detection at the genome wide level. Thus, the pediatric model picked out additional genes currently not identified in the adult setting. Regarding function, mouse models have demonstrated a role for *OLFM4* in the host gastric mucosa immune response against *H. pylori* infection,^136^ while HOXB5 is a member of the homeobox transcription factor family and has been implicated in gut development and fat loss.^137^,^138^ Therefore, these recent findings point to a possible role of the gut in determining BMI in early childhood and beyond.

## Missing heritability

Since GWAS came on the scene in late 2005, a number of loci have been found for obesity, and, importantly, there is now strong consensus in the field that these associations are robust. The current challenge is to understand the underlying biologic mechanisms of these loci and how they confer risk for obesity; indeed, almost all of the SNPs capturing association in a GWAS outcome are not causal, rather they are tagging a causative event in the proximity of the signal so those key events have still also to be elucidated.

Another major challenge is that the design of the GWAS approach is based on the common disease, common variant hypothesis, where it is postulated that the genetic component to complex diseases is largely attributable to a moderate number of common variants, each of which only explain a small proportion of risk in a population. Many loci have been identified for obesity, yet very little of the apparent heritability has been explained. Typically about 10% of the estimated heritability has been explained for most complex traits when applying GWAS; indeed, the strongest obesity-associated variants in *FTO* and *MC4R* only account for less than 2% of the variance in adult BMI, with the combined results of all obesity GWAS loci still accounting for only a very small fraction of the heritability of BMI.^123^,^139^ As such, there is a great deal of debate on what the missing heritability of most complex traits consists of.^140^ The main hypothesis right now is that it is made up of much rarer variants, copy number variants and epigenetic changes that are not detected within the bandwidth of GWAS.^66^

There is, of course, the counter argument that the estimates of missing heritability are incorrect. Indeed, Zuk *et al.*^141^ suggested that the estimated missing heritability not picked up by GWAS could be hugely overinflated, as the assumption is that the genetic variants uncovered to date do not interact with each other. Even if they interact modestly, then missing heritability estimates could be somewhat different from current understanding.

Despite this debate, it is clear there are more variants to uncover in the genetics of obesity, and some of the new approaches to find them are outlined below.

## Rare SNPs

The current commercially available genotyping arrays products are generally based on the HapMap and only provide comprehensive coverage of common variants with a minor allele frequency greater than 5%; although it should be noted that newer chips do go down to 1% frequency based on information from the 1000 Genomes project but this list is far from exhaustive. Therefore, GWAS cannot generally capture association with rare variants with minor allele frequency less than 5%. For example, one rare SNP, rs10487818, within the intron 4 of *NAMPT* has been implicated in patients with severe obesity;^142^ such a variant would not be detected by current genotyping methods. Therefore, genotyping array products will have to be much more comprehensive in genomic coverage or very large sample sizes will need to be whole genome sequenced in order to determine the remaining genetic component of obesity, the latter of which still remains somewhat cost prohibitive.

## Copy number variation

Copy number variants (CNVs) are products of genomic rearrangements, resulting in deletions, duplications, inversions, and translocations. The most established CNV in the obesity field is a large, rare chromosomal deletion of at least 593 kb at 16p11.2. Heterozygotes for this event have been shown to be significantly enriched in Caucasian patients with severe early-onset obesity and developmental delay.^143^,^144^ These deletions, albeit very rare, have been shown not to be present in healthy nonobese controls but in 0.7% of morbidly obese cases (BMI ≥40 kg/m^2^), yielding an impressive odds ratio of 43.0. Bochukova *et al.*^145^ showed that *SH2B1* is the common gene to all deletions detected and, interestingly, this gene product plays a role in leptin and insulin signaling plus energy homeostasis;^145^ in addition GWAS have revealed common variation near this locus,^123^,^145^ thus making it a target of high interest going forward.

Using the same definition of obesity used in the GWAS of childhood obesity (BMI ≥95th percentile), the same study group also addressed CNVs for this phenotype.^146^ Again, leveraging a European American cohort was used to discover any associated events but then it was asked if any detected variants replicated in a completely different ethnicity, for example, African Americans. A total of 17 CNV loci were identified that were exclusive to our European American cases and were unique to the study cohort. These were then tested in the independent African American cohort, and, surprisingly, eight of these loci, that is, approximately half, were also exclusive to cases in this ethnicity (6 deletions affecting *EDIL3, S1PR5, FOXP2, TBCA, ABCB5*, and 2 duplications affecting *KIF2B* and *ARL15*). A deletion affecting the *EPHA6-UNQ6114* locus was also implicated when the AA cohort was used as the discovery dataset. The majority of the genes have not been implicated in obesity before; however, *ARL15* has recently been detected in a GWAS of adiponectin levels, with the same risk allele being associated with a higher risk of CVD and T2D.^147^

The search for CNVs in the context of obesity has proved fruitful and it has become quite clear they play a role in the missing heritability that still needs to be explained for the disease.

## Epigenetics

Epigenetics is the study of heritable changes in gene expression or cellular phenotype caused by mechanisms other than changes in the underlying DNA sequence. These changes include DNA methylation and histone modification, both of which regulate gene expression without altering the linear sequence of DNA.

Prader–Willi syndrome exhibits developmental defects, cognitive disabilities, excessive eating and life-threatening obesity. It is an imprinting disorder that is caused by genetic and epigenetic errors in the region of chromosome 15q11-q13. The paternal copy in this region is deleted, while the maternal copy is inactivated by methylation.^148^,^149^ In addition, a genome-wide linkage analysis from African American, European American, and German populations identified several obesity-related genetic loci with differing parental effects or maternal effects.^150^ Furthermore, the pattern of DNA methylation at the human leptin promoter varies between alleles and cells, so it is possible that imprinting is locus and cell type specific.

Such observations suggest that BMI is also partially regulated by epigenetic mechanisms, which have still to be fully elucidated. New techniques, such as ChIP-Seq and whole genome sequencing, should be able to provide us with a more detailed picture of global methylation and histone modification in the future.

## Conclusions

In the past two decades, family studies and animal models have helped us to identify many genetic events associated with obesity. Subsequently, GWAS have driven the transition from primarily studying monogenic traits to ones of a more polygenic nature. GWAS have also revolutionized the genomics of obesity field, in that it offers an unbiased approach to uncover novel common genetic variants contributing to the pathogenesis of obesity.

Despite these great advances, the combined results of linkage, candidate gene, and GWAS approaches have explained very little of the variance in BMI, suggesting that there are still many genetic findings to be made, most likely being rarer variants exhibiting small effects. In order to fully characterize this missing heritability, larger and larger sample sizes are going to be required to improve statistical power. New technologies, such as next generation sequencing, will help us identify these elusive obesity-associated variants, particularly as the price of these techniques continues to drop.

In addition, most of variants that capture the association with obesity from current GWAS are not themselves causative. As such, how to move from association to causality remains a big challenge for common complex diseases like obesity. Therefore, we need to develop new approaches for analysis to characterize the true causative genes and perform functional studies to determine their roles in obesity.

Once at least some of these challenges have been mastered, we will have a clearer picture of the genomics of obesity. This, in turn, will help us produce more efficacious therapies and will guide us on the path to personalized medicine.
